# Working at home and expectations of being available: effects on perceived work environment, turnover intentions, and health

**DOI:** 10.5271/sjweh.3996

**Published:** 2022-02-25

**Authors:** Stein Knardahl, Jan Olav Christensen

**Affiliations:** 1 National Institute of Occupational Health, Oslo, Norway

**Keywords:** control, expectation being available to employer, intention to leave, job demand, mental distress, neck pain, organizational commitment, role conflict, social support, work at home

## Abstract

**Objectives:**

The aim of this study was to determine if (i) working at home and (ii) expectations of being available to the employer in their spare time influences employees’ perceptions of their work environment and well-being, health, organizational commitment, or intention to leave.

**Methods:**

We conducted cross-sectional analyses of survey data from 7861 office workers reporting hours worked at home and 3146 reporting frequency of expectations of being available to the employer in spare time (availability expectations). Prospective analyses (two-year follow up) comprised 5258 and 2082, respectively. Dependent variables were work factors previously associated with health complaints, mental distress, positive affect, work–private life conflict, commitment, and intention to leave. Random intercept linear and logistic regressions controlled for time worked (in addition to regular hours), age, gender, and skill level.

**Results:**

“Hours working at home” was cross-sectionally associated with higher levels of demands, role ambiguity, role conflicts, decision control, empowering leadership, human resource primacy, commitment, work–private life conflict, and lower support from co-workers. “Availability expectations” was associated with higher levels of demands, role conflicts, neck pain, mental distress, thinking that work was not finished when going to bed, sleep problems, work–private life conflict, intentions to leave and with lower levels of superior support, co-worker support, fair leadership, and commitment. There were no prospective associations.

**Conclusions:**

Working at home was associated with both positive and negative factors. Specific factors pertaining to role expectations and support from co-workers pose challenges. Availability expectations was associated with potentially negative work factors and health, organizational commitment, and intentions to leave. There were no long-term effects.

Telework was originally proposed in the 1970s as a means to reduce pollution and distress from commuting. Information and communication technology (ICT) with the possibility to access data from remote locations and video conferencing has allowed alternative ways of organizing work (1). The COVID-19 pandemic with restrictions on public commuting and social interactions has been a catalyst of implementation of remote work in employees’ homes, and it seems reasonable to expect that working at home will constitute a significant part of working life for a large number of people in the future (2). The overarching aims of the present study were to determine if (i) working at home and (ii) expectations of being available to the employer in their spare time influences (a) employees’ appraisal of key psychosocial work factors and (b) organizational commitment, turnover intentions, well-being, or health. We investigated the time period prior to COVID-19 since the imposed remote work due to the pandemic was acute, enforced, and total.

Previous reviews have included all work locations under the concepts of remote work and telecommuting (3, 4). Charalampous and co-workers (3) concluded that “there is still a greater consensus towards a beneficial impact of this working arrangement” (p69). Gajendran & Harrison (4) concluded with “small but favorable effects on perceived autonomy, work-family conflict, job satisfaction, performance, turnover intent, and stress” (p1538). Almost all previous studies of working at home or remote work have addressed work–private life conflict or general outcomes like job satisfaction, engagement, productivity, and ‘stress’. A systematic review of studies about working at home from 2010 to February 2021 (5) found only three studies of effects on work-environment characteristics [(control of decisions, co-worker support; 6, 7), perceived fairness; 8)]. Hence, there is scant knowledge of effects on employees’ appraisal of specific work factors.

Working at home may potentially influence all aspects of one’s job. While working at home may allow more autonomy and control of working hours with possibility for breaks and errands at one’s discretion, working hours may expand due to perceptions of what is expected (9). The elimination of travel to work also eliminates the delimitation of work by leaving the workplace [see work–family border theory (10) and boundary theory (11)]. Some organizations expect their employees to attend to and respond to messages in their spare time (9). We have not found studies of consequences of expectations to be available for the employer on appraisal of work environment or health or attitudes.

Working at home can challenge communication between the employee and her/his leader and with co-workers. One consequence is lower levels of social interactions (3, 4, 7), in particular face-to-face meetings. Other hypothetical consequences of interactions and communicating per distance are ambiguous or conflicting definitions of goals and standards, ie, role ambiguity and conflicts. We have previously reported that role conflicts prospectively predict neck pain, headache, mental distress, and disability retirement (12, 13, 14, 15). Furthermore, leaders’ capacity to maintain support and fair and empowering leadership and justice may be challenged. Therefore, organizing work in employees’ homes may potentially influence task-, group-, and organizational level aspects of the work environment. Hence, there is need for knowledge of a comprehensive set of work environment outcomes to support a sustainable implementation of working at home. Based on a large number of employees from private and public organizations, this study aimed to contribute new comprehensive knowledge of effects of working at home and expectations for being available to the employer in one’s spare time on perceptions of task and group level, and leadership factors that can influence health. We also elucidated effects of both factors on organizational commitment, turnover intentions, and health.

There are several reasons for working at home and studies should distinguish between working at home and working extra hours at home. Working at home may be a consequence of high levels of quantitative demands necessitating extra work, but it may also result from high (internal) motivation for the job. The present study sought to delineate working in one’s home by taking overtime work into account in the analyses.

The present study was based on data from a comprehensive multifactor full-panel prospective project with the aim to elucidate effects of new ways of working in the 21^st^ century in Norway. The data collection was initiated in 2004 and organizations were recruited *de novo* until 2019. Those organizations that took part in two survey waves with an approximately 24-month interval were included in prospective analyses.

## Methods

### Study design and population

The study was part of the project “The new workplace: work factors, sickness absence, and exit from working life” with full-panel prospective design. Organizations were recruited throughout 2004–2019, hence the first measurement survey took place within this extended period. Private and public organizations participated (municipalities, government ministries, federal agency, health care, finance, insurance, education, and non-profit organizations). All current employees of each organization were invited to participate (organizational level convenience sampling method). For those organizations that took part in two survey waves, the interval between survey waves ranged from 17 to 36 months (average 24 months, second survey within 2006–2019). The surveys were primarily web-based (ca 15% responded on a paper version). There was no information of hypotheses or research questions in the information conveyed to participants.

The Norwegian Regional Committee for Medical and Health Research Ethics (REC) and the Norwegian Data Inspectorate approved the study, which was conducted in accordance with the Declaration of Helsinki.

Two samples were defined for the current analyses, a cross-sectional sample for which all employees in companies that participated at least once were eligible and a prospective sample comprising employees from companies that participated at least twice. The cross-sectional sampling frame consisted of 26 841 invited employees of 1482 work units in 101 companies. Of these, 11 604 individuals (43.2%) provided information about whether they worked in an office, and 8086 (69.7% of 11 604) were office workers eligible for inclusion. Of these office workers, 7861 (97.2% of the office workers) provided information about hours spent working at home.

The *prospective* sampling frame comprised 15 580 invited employees of 986 work units in 69 companies. Of these, 7865 individuals provided information about office work status, 5418 were office workers (68.9% of those providing information), and 5258 provided information about hours working at home (97.0% of office workers).

Expectations of being available to employers in one’s spare time (availability expectations) was only asked of subjects who reported working at home (>0 hours). The number of workers who completed this item was 3146 for *cross-sectional* and 2082 for *prospective* analyses.

### Exposure measures

Working at home was introduced with “Many employees can work at home either by bringing work to their home or by electronic connection with internet (telework)”. Time spent working at home (hours worked at home) was assessed by an affect-neutral question: “How many hours per week did you work in your own home during the last week?” with response categories 0, 0–2, 2–5, 5–15, >15 hours.

A specific aspect of role expectations that may be relevant for new ways of working, boundary theory (6), and restitution was assessed by the question “Is it expected that you are available to your employer in your spare time?” (availability expectations) with frequency of occurrence response alternatives (five levels, “very seldom or never” to “very often or always”). Both questions were constructed for the present project.

Baseline-follow-up sample correlations for hours worked at home and availability expectations were 0.66 and 0.67, respectively (Spearman’s ρ, P<0.001).

### Outcome measures: work factors

The General Nordic Questionnaire for Psychological and Social Factors at Work (*QPSNordic*) has been extensively validated, has shown good psychometric properties (16, 17) and provides a comprehensive assessment of key work factors.

The following factors were studied (see supplementary material, www.sjweh.fi/article/3996, appendix): Task level: *quantitative demands* (time pressure, amount of work; 4 items), *decision demands* (3 items), *decision control* (5 items), *control over work pacing* (4 items), *role conflict* (3 items), *role ambiguity* (3 items); Group level: *support from co-workers* (2 items); Leadership: *support from immediate superior* (3 items), *fair leadership* (3 items), *empowering leadership* (3 items), *human resource primacy* (3 items). Cronbach’s α ranged from 0.61 for decision demands to 0.88 for empowering leadership. The two support-from-co-workers items exhibited Pearson’s r correlation 0.67.

Since most work factors may vary over time, response categories were frequency of occurrence (five levels, “very seldom or never” to “very often or always”) for all scales except *human resource primacy* (five categories, “very little or not at all” to “very much”).

### Outcome measures: well-being, health, attitudes to job

*Sleep* disturbance was recorded with two items “difficulties falling asleep” and “disturbed sleep” in the last four weeks. Response alternatives were “0”, “1–3 times per month”, “1–2 times per week”, “3–5 times per week”, and “6–7 times per week”. The two items were correlated (ρ=0.68, P <0.01).

*Neck pain* and unspecified *headache* were items of a checklist of 21 health complaints (18). Each complaints were recorded by asking “have you been troubled by …. (ie, neck pain) the last four weeks”, with four-level intensity scales. The wording “troubled by” is a common way of expressing the experience of a symptom in Norwegian. If reporting pain (“a little troubled” or stronger), the subject was asked to rate duration of the complaint (“1–5”, “6–10”, “11–14”, or “15–28 days”). Low-intensity chronic pain may be as severe a health problem as more intense pain with short duration. Therefore, intensity and duration were multiplied to form a *complaint-severity score* (range 0–16, ordinal scale).

*Mental distress* (symptoms of anxiety and depression) during the previous (“last”) week was measured by a 10-item Norwegian version of the Hopkins Symptom Checklist-10 (HSCL-10; 19, 20). Response alternatives were “not at all”, “a little”, “quite a bit”, and “extremely”. Cronbach’s alpha was 0.86.

*Positive affect* was measured with three questions from the Work Ability Index (WAI; 21; “have you been able to enjoy your regular daily activities recently?”, “have you been active and alert recently?”, “have you felt full of hope for the future recently?”). Responses were given on a five-level frequency scale (“never” to “often”). Cronbach’s alpha was 0.85.

*Working in the home is problematic for family (WaH–problem)* was assessed with one item with five-level frequency scale.

*Work–private life conflict (WPC)* was measured with two items from the QPS_Nordic_ “Do you feel that demands from the workplace interfere with your private- and family life?” and “Do you feel that demands from your private- and family life interfere with how you execute your work?” Response alternatives were five-level frequency scales. The Pearson’s r correlation between WaH–problem and WPC was 0.39.

*Organizational commitment* was assessed with three items from the QPS_Nordic_ (“I tell my friends this is a good organization to work in”, “my values are very similar to those of the organization”, “the organization inspires me to do my best”) with five response categories (“totally disagree” to “ totally agree”). Cronbach’s alpha was 0.86.

*Intention to leave* was measured by two items from the Michigan Organizational Assessment Questionnaire (MOAQ) (22; “I often think about quitting my job” and “It is likely that I will actively look for a new job during the next year”), with five response categories (“Completely disagree” to “Completely agree”). The two items were averaged and treated as a continuous outcome. The Pearson’s r correlation between these items was 0.70.

### Control variables

*Overtime work* was included as covariate in analyses since working at home may represent extra or overtime work (“How often have you worked more than the regular working hours during the last four weeks?” with five response alternatives from “Never” to “Very often”).

*Gender, age*, and *skill level* were included as covariates in all analyses. Skill levels were determined based on occupations, according to a Norwegian adaptation of the International Standard Classification of Occupations (ISCO-88), by Statistics Norway. This classification expresses educational levels or equivalent levels of work experience typically required for different occupations (13). Skill level also serves as proxy for socioeconomic status.

Several variables may be influenced by macro-economic fluctuations that affect labor markets and implementation of technology. Hence, *the year of the initial survey measurement* was included as covariate in all analyses.

### Statistical analyses

Statistical analyses were run with R version 4.0.0 (R Foundation for Statistical Computing, Vienna, Austria). Due to the high number of tests the criterion of statistical significance was P<0.01 and tables’ confidence intervals (CI) 99%.

Data were analyzed by mixed effects regressions with random intercepts to correct for potential bias due to correlated responses within work units. For outcome variables considered continuous, linear mixed models were used and for outcomes considered ordered categorical (neck pain, headache) cumulative link mixed models (ie, random intercept ordinal logistic regressions) were run. The models were estimated with the packages “lme4” (23) and “ordinal” (24). Including random effects in regression models accounts for possible nonindependence of measurements within clusters, thus correcting for potential bias due to clustering effects that may otherwise deflate standard error estimates and increase the risk of type I error.

Organizations that participated differed considerably in size and scope, with some being one unit organizations and others consisting of many work units distributed over a large geographical area. Work unit membership was considered an appropriate cluster variable, as employees within work units were generally assumed to share context to a larger degree than employees of the overarching organizations.

The prospective analyses included the respective outcome variables at the first measurement occasion as covariates. Attrition analyses showed that hours working at home, availability expectations, gender, working more than regular hours, or leadership responsibilities were not associated with dropout from the study. Age was associated with a statistically significant increased probability of responding at follow-up for all outcomes with odds ratios (OR) ranging from 1.01 to 1.02 and CI for all OR being (1.01–1.03) (analyses not shown). For positive affect and turnover intention, the skill level classified as “managers and unspecified” was associated with increased probability of responding, with OR of 1.58 (95% CI 1.14–2.18) and 1.40 (95% CI 1.00–1.96) (analyses not shown).

The GRADE (Grading of Recommendations, Assessment, Development and Evaluations) system hold that certainty of evidence can be up-graded if there is a dose–response gradient (25). Therefore, we highlighted associations that exhibited monotonic dose–response relationships.

## Results

Table 1 shows the number of respondents who work at home (39.2%. Of these, 52.9% reported availability expectations sometimes or more often and 69.7% reported thinking sometimes or more often that work was not finished when going to bed. Hours worked at home was associated (cross-sectionally) with reporting longer than regular working hours (Pearson’s r = 0.38, P<0.01).

**Table 1:** Descriptive statistics.

	N	%
Sex		
Male	3723	46.0
Female	4363	54.0
Age ^a^		
Skill level (years)		
>15	3069	38.4
13–15	2212	27.7
10–12	1537	19.3
<10	31	0.4
Managers and unspecified	1134	14.2
Time working at home (hours)		
0	4783	60.8
0–2	1285	16.3
2–5	986	12.5
5–15	660	8.4
>15	147	1.9
Problematic for family situation		
Very seldom or never	2086	67.5
Rather seldom	592	19.2
Sometimes	361	11.7
Rather often	41	1.3
Very often or always	10	0.3
Not finished by bedtime		
Very seldom or never	397	12.7
Rather seldom	553	17.6
Sometimes	1163	37.1
Rather often	763	24.3
Very often or always	262	8.3
Expectations to be available (availability expectations)		
Very seldom or never	857	27.2
Rather seldom	623	19.8
Sometimes	957	30.4
Rather often	378	12.0
Very often or always	331	10.5
Worked more than regular hours		
Very seldom or never	1695	21.2
Rather seldom	1678	21.0
Sometimes	2321	29.1
Rather often	1596	20.0
Very often or always	688	8.6

a Mean 44.5 (standard deviation 10.4) years

### Work factors

In order to elucidate effects or working at home *per se*, the following analyses were adjusted for reporting longer working hours, gender, age, and skill level. *Hours worked at home* was associated (cross-sectionally) with reporting higher quantitative demands, decision demands, expectations of availability, role ambiguity, role conflicts, control of decisions, empowering leadership, and human resource primacy (supplementary material table S6). The association with availability expectations reflected a monotonic dose–response relationship, while those of the two job demands factors seemed to level off at 5–15 hours per week. The other significant associations reflected threshold effects (role ambiguity, empowering leadership, human resource primacy).

*Hours working at home* was associated (cross-sectionally) with reporting lower support from co-workers, with a monotonic dose–response pattern (table 6).

*Availability expectations* was positively associated with quantitative demands, decisions demands, and role conflicts with monotonic dose–response relationships, while a significant association with role ambiguity showed no dose–response relation (table 2). *Availability expectations* was negatively associated with support from both superior and fair leadership with a monotonic dose–response relationships. Availability expectations was also associated with reporting lower support from co-workers.

**Table 2:** Cross-sectional associations between expectation of being available to employer in spare time (availability expectations) and work environment (separate linear random intercept regressions with being available to employer in spare time as predictor and work factors as outcomes). [CI=confidence interval.]

	Demand Quantity	Demand Decision	Control Decision	Control Intensity	Support from supervisor	Support from coworkers	Role ambiguity	Role conflict	Empowering leader	Fair leader	Human Resource Primacy
										
	b-value (99% CI)	b-value (99% CI)	b-value (99% CI)	b-value (99% CI)	b-value (99% CI)	b-value (99% CI)	b-value (99% CI)	b-value (99% CI)	b-value (99% CI)	b-value (99% CI)	b-value (99% CI)
Expect available spare time											
Very seldom or never	Reference	Reference	Reference	Reference	Reference	Reference	Reference	Reference	Reference	Reference	Reference
Rather seldom	0.11^c^ (0.03–0.20)	0.05 (-0.03–0.14)	0.01 (-0.08–0.11)	0.05 (-0.05–0.16)	-0.08 (-0.21–0.05)	-0.10 ^a^ (-0.21–0.01)	0.19 ^c^ (0.08–0.29)	0.05 (-0.05–0.15)	0.04 (-0.10–0.17)	-0.08 (-0.19–0.04)	0.03 (-0.08–0.14)
Sometimes	0.23 ^c^ (0.15–0.31)	0.09 ^b^ (0.01–0.17)	0.03 (-0.05–0.12)	0.03 (-0.07–0.13)	-0.15 ^b^ (-0.27– -0.03)	-0.19 ^c^ (-0.29– -0.08)	0.13 ^c^ (0.03–0.22)	0.20 ^c^ (0.10–0.29)	0.04 (-0.09–0.17)	-0.13 ^b^ (-0.24– -0.02)	-0.02 (-0.13–0.09)
Rather often	0.33 ^c^ (0.23–0.43)	0.18 ^c^ (0.07–0.29)	-0.07 (-0.18–0.05)	0.02 (-0.12–0.16)	-0.25 ^c^ (-0.41– -0.09)	-0.20 ^c^ (-0.34– -0.06)	0.14 ^b^ (0.01–0.26)	0.26 ^c^ (0.13–0.39)	-0.04 (-0.21–0.13)	-0.28 ^c^ (-0.42– -0.13)	-0.08 (-0.22–0.06)
Very often or always	0.41 ^c^ (0.30–0.53)	0.30 ^c^ ( 0.18–0.42)	-0.03 (-0.16–0.10)	-0.03 (-0.18–0.12)	-0.33 ^c^ (-0.50– -0.15)	-0.18 ^b^ (-0.33– -0.03)	0.06 (-0.08–0.20)	0.33 ^c^ (0.19–0.47)	-0.11 (-0.30–0.08)	-0.34 ^c^ (-0.50– -0.19)	-0.12 ^a^ (-0.28–0.03)

a P<0.05.

b P<0.01.

c P<0.001.

### Well-being, health, attitudes to job

*Cross-sectional associations*: *Hours working at home* was associated with higher levels of organizational commitment, with thinking that work was not finished when going to bed, and with WPC (table 3), the latter two showed monotonic dose–response relationships.

**Table 3:** Cross-sectional associations between time working at home (WaH) (Hours worked at home) and well-being, health complaints, and turnover intentions. [OR=odds ratio; CI=confidence interval.]

	Neck pain	Headache	Mental distress	Not done with work	Sleep problems	Positive affect	WaH problems ^b^	Work–Home Conflict	Org. commitment	Intention to leave
	
	OR (99% CI)	OR (99% CI)	b-value (99% CI)	b-value (99% CI)	b-value (99% CI)	b-value (99% CI)	b-value (99% CI)	b-value (99% CI)	b-value (99% CI)	b-value (99% CI)
Time WaH (hours)										
0	Reference	Reference	Reference	Reference	Reference	Reference	Reference	Reference	Reference	Reference
0–2	0.93 (0.77–1.12)	1.05 (0.88–1.26)	0.01 (-0.03–0.04)	0.17 (-0.22–0.56)	-0.01 (-0.10–0.08)	0.02 (-0.05–0.09)	-0.11 -0.46–0.23)	0.16 ^c^ (0.10–0.23)	0.15 ^c^ (0.07–0.22)	-0.02 (-0.13–0.08)
2–5	0.89 (0.71–1.11)	1.05 (0.85–1.28)	0.02 (-0.02–0.07)	0.44 ^b^ (0.04–0.83)	0.09 ^a^ (-0.01–0.19)	0.07 ^a^ (-0.01–0.16)	-0.02 (-0.37–0.33)	0.29 ^c^ (0.22–0.36)	0.16 ^c^ (0.07–0.25)	-0.07 (-0.20–0.05)
5–15	0.82 (0.63–1.07)	1.16 (0.92–1.47)	0.01 (-0.04–0.06)	0.53 ^c^ (0.13–0.93)	0.02 (-0.10–0.14)	0.08 ^a^ (-0.02–0.18)	0.01 (-0.34–0.36)	0.37 ^c^ (0.28–0.46)	0.19 ^c^ (0.08–0.29)	-0.02 (-0.17–0.13)
>15	0.65 ^a^ (0.38–1.11)	1.00 (0.61–1.65)	0.03 (-0.08–0.13)	0.68 ^a^ (0.23–1.13)	-0.01 (-0.25–0.23)	0.09 (-0.10–0.29)	0.02 (-0.36–0.41)	0.51 ^c^ (0.34–0.68)	0.19 ^a^ (-0.01–0.40)	-0.07 (-0.36–0.23)

a P<0.05.

b P<0.01.

c P<0.001.

*Availability expectations* was positively associated with neck-pain, mental distress, thinking that work was not finished when going to bed, sleep problems, problems for family situation, and WPC with dose–response relationships (table 4). *Availability expectations* was negatively associated with commitment and positively associated with turnover intentions.

**Table 4:** Cross-sectional associations between expectations of being available in spare time (availability expectations) and well-being, health complaints, and turnover intentions. [WaH=working at home.]

	Neck pain	Headache	Mental distress	Not done with work	Sleep problems	Positive affect	WaH Problems	Work –Home Conflict	Org. commitment	Intention to leave
									
	OR (99% CI)	OR (99% CI)	b-value (99% CI)	b-value (99% CI)	b-value (99% CI)	b-value (99% CI)	b-value (99% CI)	b-value (99% CI)	b-value (99% CI)	b-value (99% CI)
Expect available spare time										
Very seldom or never	Reference	Reference	Reference	Reference	Reference	Reference	Reference	Reference	Reference	Reference
Rather seldom	0.94 (0.70–1.25)	1.00 (0.75–1.33)	0.07 ^b^ (0.01–0.13)	0.33 ^c^ (0.19–0.47)	0.12 ^a^ (-0.02–0.26)	-0.05 (-0.16–0.06)	0.07 (-0.03–0.18)	0.19 ^c^ (0.08–0.29)	-0.03 (-0.14–0.09)	0.04 (-0.13–0.22)
Sometimes	1.28 ^a^ (0.98–1.67)	1.13 (0.87–1.47)	0.07 ^b^ (0.01–0.12)	0.40 ^c^ (0.27–0.54)	0.11 ^a^ (-0.02–0.24)	-0.08 (-0.18–0.03)	0.14 ^c^ (0.04–0.24)	0.30 ^c^ (0.21–0.40)	-0.12 ^b^ (-0.23,-0.01)	0.15 ^a^ (-0.01–0.31)
Rather often	1.48 ^b^ (1.04–2.11)	1.21 (0.85–1.73)	0.11 ^c^ (0.04–0.19)	0.56 ^c^ (0.38–0.74)	0.15 ^a^ (-0.03–0.32)	-0.14 ^a^ (-0.28–0.01)	0.22 ^c^ (0.09–0.36)	0.41 ^c^ (0.28–0.54)	-0.20 ^c^ (-0.35,-0.05)	0.32 ^c^ (0.11–0.54)
Very often or always	1.50 ^b^ (1.02–2.23)	1.43 ^a^ (0.97–2.13)	0.12 ^c^ (0.04–0.20)	0.72 ^c^ (0.52–0.91)	0.31 ^c^ (0.12–0.50)	-0.15 ^a^ (-0.30–0.01)	0.31 ^c^ (0.16–0.45)	0.46 ^c^ (0.33–0.60)	-0.22 ^c^ (-0.38,-0.06)	0.29 ^b^ (0.06–0.53)

a P<0.05.

b P<0.01.

c P<0.001.

*Note: All regressions were adjusted for working more than regular hours, gender, age, skill level, leader/management responsibility, and year of measurement. Estimates are fixed effects from random coefficient regressions. For neck pain and headache, cumulative link mixed models with random intercepts were run and odds ratios are presented, for the remaining factors linear mixed models with random intercepts were run and b-values are presented.*

*Prospective associations (two-year follow up)*: neither *hours working at home* nor *availability expectations* showed statistically significant prospective associations with any of the measured indicators of well-being, health or attitudes to the job (see table 5 and supplementary material: table S7).

**Table 5:** Prospective associations between expectations of being available in spare time (availability expectations) and well-being, health complaints, and turnover intentions. [OR=odds ratio; CI=confidence interval.]

	Neck pain	Headache	Mental distress	Sleep problems	Positive affect	Organizational commitment	Intention to leave
						
	OR (99% CI)	OR (99% CI)	b-value (99% CI)	b-value (99% CI)	b-value (99% CI)	b-value (99% CI)	b-value (99% CI)
Expect available spare time							
Very seldom or never	Reference	Reference	Reference	Reference	Reference	Reference	Reference
Rather seldom	1.21 ( 0.78–1.87)	1.13 ( 0.73–1.76)	-0.02 (-0.08–0.05)	-0.04 (-0.20–0.11)	0.00 (-0.15–0.14)	0.01 (-0.13–0.15)	0.09 (-0.12–0.29)
Sometimes	1.21 ( 0.81–1.83)	1.09 ( 0.72–1.65)	0.02 (-0.04–0.08)	-0.03 (-0.18–0.11)	-0.03 (-0.16–0.11)	-0.09 (-0.22–0.04)	0.12 (-0.06–0.31)
Rather often	1.49 ( 0.88–2.53)	1.23 ( 0.71–2.14)	0.05 (-0.03–0.13)	0.09 (-0.11–0.28)	-0.07 (-0.25–0.11)	-0.06 (-0.23–0.11)	0.11 (-0.14–0.36)
Very often or always	1.06 ( 0.56–2.01)	1.13 ( 0.61–2.09)	0.00 (-0.09–0.09)	-0.12 (-0.33–0.10)	-0.03 (-0.23–0.17)	-0.03 (-0.21–0.16)	0.18 (-0.09–0.46)

Note: Analyses were adjusted for working more than regular hours, gender, age, skill level, leader/management responsibility, and year of measurement.

## Discussion

The present study of office workers was undertaken prior to the COVID-19 pandemic and the prevalence of working at home was moderate (39.2%). Reporting that they sometimes or more often were expected to be available for work in leisure time (availability expectations) was common (52.9%). Hours working at home showed associations with both quantitative- and decision *demands* (table 6). Availability expectations was associated with reporting higher levels of both types of *job demands* (table 2) with monotonic dose–response relationships. Since the present analyses included overtime work as a covariate, one should expect that the association between hours working at home and the perception of higher job demands reflect aspects of working in one’s home rather than having longer working hours. This finding is in accordance with a representative study from the UK that reported “more voluntary effort is expended” among *remote* workers (26, p205), but contrasts with reports of lower work effort in the 3.4% of US federal agency employees who teleworked (27). There are several possible explanations for associations between working at home and job demands. Working at home may be an inherent part of the job or a coping response to high levels of demands (eg, 28). Working at home may also reflect internal motivation for job tasks (involvement, 29) and there was a positive association between hours working at home and commitment (table 3). Moreover, it is possible that hours working at home and availability expectations increase the perception of job demands by making the employee think about the job for larger parts of the day. Indeed, both factors were associated with thinking that the job was not done when going to bed.

Hours working at home and particularly availability expectations were associated with *role ambiguity* and *role conflicts*. Previous studies have reported mixed results. Telework was negatively related to role conflict and positively related to role ambiguity among US teleworking supply chain management employees (30), and US teleworking federal agency employees reported lower role ambiguity (27). The present finding may be a result of lower levels of supervision of employees working at home. Indeed, there was a negative association between availability expectations and reported support from one’s superior and with fair and empowering leadership. An alternative explanation is that employees who experience negative work factors cope by doing some of the work in their homes (ie, reverse causality).

Hours working at home and availability expectations were associated with lower *coworker support*. This is in accordance with a study of homework of employees of a Belgian telecommunications company (7). Promoting optimal levels of social interactions and preventing social isolation are challenges for organizations implementing remote work.

One notable finding was that hours working at home was positively associated with perceived *empowering leadership* and *control of decisions*, while availability expectations showed negative associations with these factors.

Similarly, hours working at home and availability expectations showed opposite associations with level of *organizational commitment*: Hours working at home promoted while availability expectations attenuated commitment. The positive effects of working at home on job control and empowerment may promote commitment. On the other hand, it is conceivable that higher level of commitment motivates working at home (resulting in more hours working at home). Expectations to be available in one’s spare time seems perceived as negative and consequently attenuates commitment. A reverse association – that lower commitment causes expectations of being available in one’s spare time – seems unlikely. The potentially negative effect of availability expectations was emphasized by its association with *intentions to leave the job*.

Both hours working at home and availability expectations were associated with *thinking that the job was not done when going to bed*. Availability expectations was also associated with *sleep problems*. Both hours working at home and availability expectations were associated with *work–private life conflict*, but only availability expectations was associated with reporting that working at home was *problematic for the family situation*. Arlinghaus & Nachreiner (31) analyzed large-scale cross-sectional surveys (Ns >22 000) and found that *supplemental* work at home (ie, working in one’s free time) was associated with self-reported health impairment. They concluded that “in order to minimize negative health effects, availability requirements for employees outside their regular work hours should be minimized” (p1100). An experimental study of being required to be available during nonworking hours showed “significant effects of extended work availability on the daily start-of-day mood and cortisol awakening response” (32).

Availability expectations, but not hours working at home, was associated with *neck pain* and *mental distress*. We have previously reported that role conflicts predicted risk of neck pain while empowering leadership attenuated the risk (12). We also found that role conflict predicted risk of mental distress while support from one’s superior and fair leadership attenuated risk (14). It seems reasonable to conclude that working at home *per se* implies both positive and negative work factors that often times cancel each other. On the other hand, availability expectations seem associated with potentially negative work factors.

There were no statistically significant prospective effects at two years follow-up. Well-being, mental health, sleep, and attitudes to one’s job vary over time and latency from exposure to response may range from hours to months. It seems that hours working at home and availability expectations vary over time or that effects are either transient or moderate. We do not have data pertaining to the duration of periods spent working at home, but correlations between the two survey-wave measurements were 0.66 and 0.67. Previous meta-analyses have suggested an optimal time-lag of 2–3 years for detecting occupational stressor-strain associations (33).

### Methodological considerations.

Many studies have analyzed working at home as a dichotomous variable. The present study graded hours of working at home and availability expectations and took frequency of overtime work into account in all analyses. Presumably, working at home was not confounded with overtime work. Single-item measures of concrete factors can exhibit high validity and reliability (34). Furthermore, the questionnaire for psychological and social work factors (QPS_Nordic_) has been thoroughly validated (12–17). The present study comprised a large number of employees reporting that they worked in an office and performed both cross-sectional and prospective analyses.

The data were collected from 2004–2020. The implementation of ICT may have changed the contents of many office jobs in this period. We sought to attenuate this problem by adjusting all analyses for year of data collection. However, one cannot eliminate the possibility that the meaning of work concepts may be transformed with digitalization of tasks. On the other hand, with this long data-collection period, transient effects of business cycles were attenuated.

The number of employees working at home >15 hours per week was low (1.9%), hence the present study cannot generalize to contexts of full-time work at home (eg, during the COVID-19 pandemic). Future arrangements of working >2 days at home (ie, >15 hours) were not adequately represented in these data. However, the findings pertaining to effects of availability expectations should be relevant to work-spare time boundaries in general.

A concern with subjective reports is the risk of method bias, ie, method factors that influence the subject’s responding, introducing method variance and/or bias of estimates of the constructs that are measured. Personality characteristics influence perception and appraisal and the reporting of exposures, situations, states, and symptoms. Neuroticism predisposes for reporting mental and somatic symptoms (eg, 35, 36). Social-desirability may produce bias by systematic over- or underreporting according to social norms (eg, 37). Response styles and heuristics to minimize cognitive effort may influence responding. Context at the time of reporting may influence affective state, situation models, and cognitive representation. However, Askim & Knardahl (38) found that transient affect has little influence on responding to neutral-valence worded questions, with the possible exception of questions measuring attitudes or social interactions. Perception and appraisal are coping mechanisms that play a role in the causal pathway for factors that contribute to motivation, well-being, health, or function in individuals. Hence, subjective appraisal is a mediator in causal processes rather than an error. The present study sought to elucidate employees’ subjective appraisal of their work situation, attitudes, and subjective health indicators.

The assumption that associations based on same-source, self-reported data are inherently invalid due to common method variance (CMV) has received much attention (39). Fuller and coworkers found that “relatively high levels of CMV must be present to bias true relationships among substantive variables at typically reported reliability levels” (40, p3192). The instruments of this study should be rather insensitive to method bias and CMV since the QPS_Nordic_-items are worded for neutral valence, respondents reported frequency of occurrence rather than degree of agreement or satisfaction, and items did not address issues that are inherently negative or positive. However, one cannot eliminate effects of personality traits.

Cross-sectional analyses elucidate short-term effects with the limitation that direction of effects cannot be ascertained, and simultaneity cannot be eliminated. For instance, the association between working at home and job demands may be bidirectional.

### Concluding remarks

The present study shows contrasting effects of two aspects of working at home. The results suggest that conducting a moderate part of working hours at home is associated with higher levels of control, empowering leadership, and commitment, ie, aspects that are positive for well-being and health. Higher levels of demands, specific role expectations, and support from co-workers pose challenges. In contrast, expectations to be available to the employer in one’s spare time seem to be associated with a series of potentially negative work factors and consequences for health, organizational commitment, and intentions to leave in the short term. The present study identifies specific effects of two aspects of working at home and should be helpful in advancing knowledge of specific factors that can be modified or prevented.

## Supplementary material

Supplementary material

## Protection of research participants

Questionnaire responses were collected with a two-stage identity-code system to ensure that participants’ responses were protected from access from other persons both during administration of questionnaires in organizations and during data storage and analysis. All participants allowed the use of the data for the present set of analyses.
