# Supplementary material for: Working at home and expectations of being available: effects on perceived work environment, turnover intentions, and health
Source: Scand J Work Environ Health. 2022 Feb 25;48(2):99–108. doi: 10.5271/sjweh.3996 (PMC9045229; doi:10.5271/sjweh.3996)
Supplement: Supplementary material [file SJWEH-48-99-S001.pdf]

Working at home and expectations of being available: effects on perceived work environment, turnover intentions, and health[1]

by Stein Knardahl, PhD, MD,[2] Jan Olav Christensen, PhD

1. *SUPPLEMENTARY MATERIAL*
2. *Correspondence to: Stein Knardahl, National Institute of Occupational Health, Post box 5330, 0304 Oslo, Norway. [E-mail: stein.knardahl@stami.no]*

Table 6. Cross-sectional associations between time working at home (Hours worked at home) and perceptions of work and work environment (separate linear random intercept regressions with Hours worked at home as predictor and work factors as outcomes).

**Time WaH**	Demand Quant	Demand Decision	Control Decision	Control Intens	Support Supervis	Support Cowork	Role Ambig	Role Conflict	Empow Leader	Fair Leader	Human Res Pri	Expect Avail
0 hrs	ref [99% CI]	ref [99% CI]	ref [99% CI]	ref [99% CI]	ref [99% CI]	ref [99% CI]	ref [99% CI]	ref [99% CI]	ref [99% CI]	ref [99% CI]	ref [99% CI]	ref [99% CI]
0-2 hrs	0.10*** [0.04, 0.15]	0.06** [0.00, 0.12]	0.18*** [0.12, 0.25]	0.06* [-0.01, 0.13]	0.05 [-0.03, 0.13]	-0.05 [-0.12, 0.02]	0.07** [0.01, 0.14]	0.05* [-0.01, 0.12]	0.11*** [0.03, 0.20]	0.03 [-0.04, 0.11]	0.12*** [0.05, 0.19]	0.24 [-0.18, 0.66]
2-5 hrs	0.15*** [0.09, 0.21]	0.06* [-0.00, 0.13]	0.24*** [0.17, 0.31]	0.04 [-0.04, 0.12]	-0.02 [-0.12, 0.07]	-0.09** [-0.17,-0.01]	0.09** [0.01, 0.16]	0.10*** [0.03, 0.18]	0.14*** [0.04, 0.24]	-0.02 [-0.10, 0.07]	0.11*** [0.02, 0.20]	0.41* [-0.01, 0.83]
5-15 hrs	0.22*** [0.15, 0.30]	0.12*** [0.04, 0.19]	0.27*** [0.19, 0.36]	0.06 [-0.04, 0.15]	0.01 [-0.11, 0.12]	-0.10** [-0.20,-0.01]	0.03 [-0.06, 0.12]	0.06 [-0.03, 0.15]	0.16*** [0.04, 0.28]	0.02 [-0.08, 0.12]	0.16*** [0.06, 0.26]	0.38* [-0.05, 0.81]
>15 hrs	0.22*** [0.07, 0.36]	0.10 [-0.05, 0.25]	0.26*** [0.09, 0.43]	0.17* [-0.02, 0.36]	0.01 [-0.20, 0.23]	-0.16* [-0.34, 0.02]	-0.05 [-0.22, 0.12]	0.11 [-0.06, 0.29]	0.10 [-0.13, 0.34]	0.18* [-0.03, 0.38]	0.11 [-0.09, 0.31]	0.57** [0.09, 1.05]

*p<0.05; **p<0.01; ***p<0.001

Note: All regressions were adjusted for working more than regular hours, gender, age, skill level, leader/management responsibility, and year of measurement

Table 7. Prospective associations between time working at home (Hours worked at home) and well-being, health complaints, and turnover intentions.

**Time WaH**	Neck pain	Headache	Mental distress	Sleep problems	Positive affect	Organizational commitment	Intention to leave
0 hrs	Ref<br>[99% CI]	Ref<br>[99% CI]	Ref<br>[99% CI]	Ref<br>[99% CI]	Ref<br>[99% CI]	Ref<br>[99% CI]	Ref<br>[99% CI]
0-2 hrs	0.96<br>[0.72,1.28]	0.94<br>[0.70,1.26]	-0.02<br>[-0.06,0.02]	0.02<br>[-0.08,0.12]	0.04<br>[-0.05,0.12]	0.04<br>[-0.05,0.13]	0.00<br>[-0.13,0.12]
2-5 hrs	1.05<br>[0.75,1.47]	0.84<br>[0.60,1.17]	-0.02<br>[-0.07,0.03]	0.03<br>[-0.08,0.15]	0.05<br>[-0.05,0.15]	0.06<br>[-0.04,0.16]	-0.06<br>[-0.21,0.08]
5-15 hrs	1.19<br>[0.80,1.77]	0.89<br>[0.59,1.33]	0.00<br>[-0.05,0.06]	0.10<br>[-0.04,0.23]	0.01<br>[-0.10,0.13]	0.06<br>[-0.06,0.18]	-0.01<br>[-0.18,0.17]
>15 hrs	0.95<br>[0.41,2.20]	0.69<br>[0.28,1.74]	0.04<br>[-0.08,0.15]	0.20<br>[-0.09,0.48]	-0.09<br>[-0.33,0.16]	-0.09<br>[-0.33,0.15]	0.17<br>[-0.19,0.53]

Note: Analyses were adjusted for working more than regular hours, gender, age, skill level, leader/management responsibility, and year of measurement. Confidence intervals are 99%.

Estimates are fixed effects from random coefficient regressions. For neck pain and headache, cumulative link mixed models with random intercepts were run and odds ratios are presented, for the remaining factors linear mixed models with random intercepts were run and b-values are presented.

APPENDIX

The selection of variables was motivated by the aim to provide new knowledge of practical relevance, i.e. of potential impact on risk analysis, prevention, and intervention. Therefore, it was considered necessary to measure a rather comprehensive selection of work factors that may be affected by working at home and availability expectations, including task-level and group-level factors as well as leadership (see Knardahl 2020 for a brief reviews of relevant theories).

Task-level factors

Job demands is what is required of the employee for performing his or her job. Hence, job demands is a broad dimension encompassing (a) amount of work, rate of work or perceived time pressure (quantitative demands), (b) demands for making decisions (decision demands), solving problems, meeting quality standards, performing difficult tasks (qualitative demands), (c) learning new skills and knowledge (learning demands), (d) social-interaction demands (often labelled emotional demands), (e) demands to manage risk and safety, and (f) demands to apply physical effort (physical demands).

European Foundation for the Improvement of Living and Working Conditions (2007). 'Work-related stress' (Retrieved 11 February 2013, from www.eurofound.europa.eu) listed four types of demands: (a) quantitative demands (e.g. time pressure or the amount of work); (b)cognitive demands that impinge primarily on the brain processes involved in information processing (e.g. the difficulty of the work); (c) emotional demands which refer primarily to the effort needed to deal with organizationally desired emotions during interpersonal transactions; or (d) physical demands that are primarily associated with the musculoskeletal system (i.e. motoric and physical aspects of behaviour).

Previous studies have reported inconsistent associations between levels of job demands and health (e.g. Theorell et al, 2015) and mental health may influence the appraisal of job demands resulting in reverse associations (Dalgard et al, 2009). Furthermore, high occupational level (employment grade) are often associated with high levels of demands, hence level of education and salary may moderate appraisal and effects of demands (e.g. North et al, 1996). A large body of evidence show that the combination of high levels of job demands and low levels of control (see below) over his or her work contributes to risk of somatic disease (e.g. Kivimäki et al, 2012), depression (Theorell et al, 2015), and disability retirement (Knardahl et al, 2017).

The current study tested the hypotheses that working at home and expectations to be available to the employer in one's spare time influenced both quantitative and qualitative aspects of demands, hence we measured *quantitative* demands, referring to amount of work and pace, and *decisional* demands, an aspect of qualitative demands which refers to having to make quick and complex decisions as well as demands for attention.

Quantitative demands was measured with the following questions (Dallner et al., 2000):

1. Is your work load irregular so that the work piles up?

2. Do you have to work overtime?

3. Is it necessary to work at a rapid pace?

4. Do you have too much to do?

Decision demands was measured with the following questions (Dallner et al., 2000):

1. Does your work require quick decisions?

2. Does your work require maximum attention?

3. Does your work require complex decisions?

Role ambiguity refers to ambiguous information and definitions of goals, responsibilities, tasks, or standards.

Role conflict refers to situations when two or more expectations are incompatible, either incompatible requests or objectives or between work and personal values and norms (Rizzo et al, 1970). Role conflict is treated as a job demand in the frequently employed Job Content Questionnaire (JCQ; Karasek et al, 1998).

Role conflicts may increase risk of pain complaints (Christensen & Knardahl, 2010; Christensen & Knardahl, 2012), mental distress (Finne et al, 2014), sickness absence (Indregard et al, 2016), and disability retirement (Emberland et al, 2017).

Working at home may challenge and constrain interactions and communication with leaders and co-workers, possibly deteriorating the process of defining roles. The present study tested the hypotheses that working at home and expectations to be available to the employer in one's spare time contributes to *role ambiguity* and/or *role conflict* (Beehr et al., 1976; Kahn et al., 1964).

Role ambiguity was measured with the following questions (Dallner et al., 2000):

1. Have clear, planned goals and objectives been defined for your job?

2. Do you know what your responsibilities are?

3. Do you know exactly what is expected of you at work?

Role conflict was measured with the following questions (Dallner et al., 2000):

1. Do you have to do things that you feel should be done differently?

2. Are you given assignments without adequate resources to complete them?

3. Do you receive incompatible requests from two or more people?

In general, *job control* refers to having the possibility or freedom to choose between alternatives (i.e. the availability of alternatives). *Job control* is a dimension that encompasses several factors, (a) control of decisions pertaining to one's job, (b) control of pace and amount of work, (c) control of procedures employed, (d) control of pauses, (e) control of social interactions, (f) control of working hours (e.g. flexitime), and (g) control of place to work. "Job autonomy" was defined by Hackman and Oldham's 'job characteristics theory' as "the degree to which the job provides substantial freedom, independence, and discretion to the individual in scheduling the work and in determining the procedures to be used in carrying it out". (Hackman & Oldham, 1975, p. 162). The Demand-Control model operationally defines job control by two separate factors - skill discretion and decision authority (Karasek, 1979; Karasek et al., 1998). Skill discretion refers to the opportunity to utilize one's skills and abilities while decision authority pertains to the freedom to make decisions, to influence and to regulate aspects of one's work such as pacing, breaks, and working hours. Important aspects are the opportunities to influence planning and decision-making relevant to one's job tasks (Dallner et al., 2000).

A large body of research has shown that low levels of control contribute to risk of health problems (e.g. Christensen & Knardahl, 2012; Finne et al, 2016; Theorell et al, 2015), sickness absence (Indregard et al, 2016), and disability retirement (see Knardahl et al, 2017 for systematic review). Most studies of effects of control have measured and analysed the broad dimension of control not attempting to elucidate specific aspects (Knardahl et al, 2017).

Working at home may imply working more autonomously, however, we have not found previous studies of working at home that elucidated control of work pace and aspects of working hours. The present study tested the hypotheses that working at home and expectations to be available to the employer in one's spare time influences level of control of decisions and control of work pacing.

Control of decisions was measured with the following questions (Dallner et al., 2000):

1. If there are alternative methods for doing your work, can you choose which method to use?

2. Can you influence the amount of work assigned to you?

3. Can you influence decisions concerning the persons you will need to collaborate with?

4. Can you decide when to be in contact with clients?

5. Can you influence decisions that are important for your work?

Control of work pacing was measured with the following questions (Dallner et al., 2000):

1. Can you set your own work pace?

2. Can you decide yourself when you are going to take a break?

3. Can you decide the length of your break?

4. Can you set your own working hours (flexitime)?

Group level: Social support

There are many dimensions of social relations. *Social integration* pertains to social ties with primary groups (i.e. persons with intimate, close, and enduring relationships: significant others like family and friends) and secondary groups (i.e. persons with more formal and less personal relationships: colleagues at work, members of voluntary organizations).

Social support pertains to either (A) the *receiving* of support or behaviours or (B) the *perception* that social support is available if needed, i.e. the sense of support. It seems that the perception of availability to receive support has the most significant influence on health and well-being (Thoits, 2011). There are two hypothetical main mechanisms of effects of social support: (i) social relationships exert a *direct* positive effect on health and (II) social support *buffers* effects of challenges, negative exposures, and distress (House et al, 1988; Thoits, 2011).

Working at home may reduce face-to-face social interactions and communication between co-workers. The present study tested the hypotheses that working at home and expectations to be available to the employer in one's spare time influences *social support from co-workers*.

Support from co-workers was measured with two questions (Dallner et al., 2000):

1. If needed, can you get support and help with your work from your co-workers?

2. If needed, is your co-workers willing to listen to your work-related problems?

Leadership

Leaders and managers are given power to influence the organization and execution of work tasks. Leadership is commonly used synonymously with management, a term derived from 'manage' to forecast and to plan, to organize, to command, to co-ordinate, and to control. On the other hand, leadership also describes the behavioural process of taking the lead, the process of social influence which leads others towards a goal or to solving a task (Knardahl, 2020). Behavioural theories of leadership focus on behaviours of leaders, and traditionally have divided leadership in two general categories: task-oriented leadership, i.e. attention to tasks that must be completed to reach a goal, and relationship-oriented leadership, i.e. attention to the motivation, satisfaction, and well-being of people (Fiedler, 1971). The present study measured employees' perceptions of a limited selection of leader behaviours that previously have been associated with well-being and health.

Level of *social support from one's superior* seems to influence many aspects of health, e.g. musculoskeletal disorders (e.g. Eriksen et al, 2004) and mental distress (Finne et al, 2014). Since working in one's home may reduce social interactions between employees and their leaders, we measured the perception of available *support from the immediate superior*. The present study tested the hypotheses that working at home and expectations to be available to the employer in one's spare time influence employees' appraisal of social support from one's immediate superior.

Support from superior was measured with the following questions (Dallner et al, 2000):

1. If needed, can you get support and help with your work from your immediate superior?
2. If needed, is your immediate superior willing to listen to your work-related problems?
3. Are your achievements appreciated by your immediate superior?

Fair leadership refers to the fairness of the immediate leader's decisions and behaviours, i.e. being unbiased and impartial and treating subordinates fairly and equally (Dallner et al., 2000). Fair leadership has been linked to lower risk of mental distress and higher levels of positive affect (Finne et al, 2016).

If channels and availability of communication is altered, leaders may find it more challenging to follow up all their employees who may receive inadequate information of the working conditions of their colleagues. Therefore, perceptions of fairness may be challenged when working at home. The present study tested the hypotheses that working at home and expectations to be available to the employer in one's spare time influences appraisal of *fair leadership*.

Fair leadership was measured with the following questions (Dallner et al, 2000):

1. Does your immediate superior distribute the work fairly and impartially?
2. Does your immediate superior treat the workers fairly and equally?
3. Is the relationship between you and your immediate superior a source of stress to you?

Human resource primacy is an indicator of organizational practices of relevance to organizational values and culture. Hence, human resource primacy pertains to the organizational level, highlighting the role of top management and the degree to which employee health and well-being are prioritized and whether priorities are communicated.

Human resource primacy has been linked to lower risk of mental distress and higher levels of positive affect (Finne et al, 2016).

Being allowed to work at home or having to work at home may be associated with the appraisal of the organization's attitudes toward employees (relation-oriented). Hence, we measured the appraisal of organizational practices pertaining to reward for well executed job tasks, taking care of employees and the interest of management in the health and well-being of employees. The present study tested the hypotheses that working at home and expectations to be available to the employer in one's spare time influence the perception of *human resource primacy*.

Human resource primacy was measured with the following questions (Dallner et al, 2000):

1. At your organization, are you rewarded (money, encouragement) for a job well done?
2. Are workers well taken care of in your organization?
3. To what extent is the management of your organization interested in the health and well-being of the personnel?

Empowering leadership refers to the delegation of responsibility and resources by encouraging employees to use and develop their competence and autonomy, to take part in decisions, and to voice their opinions (as opposed to the delegation of specific tasks) (Dallner et al, 2000). Empowering leadership may attenuate the risk of neck pain (Christensen & Knardahl, 2010).

If communication is reduced, leaders may want to promote autonomy of employees. However, with less face-to-face interactions leaders may not see practical ways to promote empowerment. In addition, with decentralized work, leaders may find that decisions are taken without consulting employees. Employees may perceive that working alone at home isolates them from taking part in decisions and developing their skills. The present study tested the hypotheses that working at home and expectations to be available to the employer in one's spare time influence the perception of empowering leadership.

Empowering leadership was measured with the following questions (Dallner et al, 2000):

1. Does your immediate superior encourage you to participate in important decisions?
2. Does your immediate superior encourage you to speak up when you have different opinions?
3. Does your immediate superior help you to develop your skills?

References (in alphabetical order):

Beehr TA, Walsh JT, & Taber TD. Relationship of stress to individually and organizationally valued states: Higher order needs as a moderator. Journal of Applied Psychology, 1976;61: 41-47.

Christensen JO & Knardahl S. Work and neck pain: A prospective study of psychological, social, and mechanical risk factors. PAIN, 2010;151:162-73.

Christensen JO & Knardahl S. Work and headache: A prospective study of psychological, social, and mechanical risk factors of headache. PAIN, 2012;153:2119-2132.

Christensen JO & Knardahl S. Work and back pain: A prospective study of psychological, social, and mechanical risk factors of back pain severity. Eur J Pain, 2012;16:921-933.

Dalgard OS, Sørensen T, Sandanger I, Nygård JF, Svensson E, Reas DL. Job demands, job control, and mental health in an 11-year follow-up study: Normal and reversed relationships. Work & Stress, 2009;23, 284-296.

Dallner M, Elo A-., Gamberale F, Hottinen V, Knardahl S, Lindström K, Skogstad A, Ørhede E Validation of the General Nordic Questionnaire (QPS_{Nordic}) for psychological and social factors at work. Nordic Council of Ministers, 2000; Nord 2000:12.

Emberland JS, Nielsen MB, Knardahl S. Psychological , social, and mechanical work exposures and disability retirement: a prospective registry study. BMC Public Health, 2017;17:56; DOI 10:1186/s12889-016-3921-0.

Eriksen W, Bruusgaard D, & Knardahl S. Work factors as predictors of intense or disabling low back pain; A prospective study of nurses′ aides. Occup Environ Med, 2004;61:398-404.

Fiedler FE. Validation and extension of the contingency model of leadership effectiveness: a review of empirical findings. Psychological Bulletin*,* 1971;*76*, 128–148.

Finne LB, Christensen JO, Knardahl S. Psychological and social work factors as predictors of mental distress: a prospective study. PLOS one, 2014; 9:e102514. Doi:10.1371/journal.pone.0102514.

Finne LB, Christensen JO, Knardahl S.. Psychological and social work factors as predictors of mental distress and positive affect: A prospective, multilevel study. PLOS one, 2016; Mar 24;11(3):e0152220. doi: 10.1371/journal.pone.0152220.

Hackman JR & Oldham GR. Development of the job diagnostic survey. Journal of Applied Psychology, 1975;60, 159-170.

House JS, Landis KR, Umberson D. Social relationships and health, Science, 1988;241, 540-545.

Indregard A-M R, Knardahl S, Nielsen MB. Emotional dissonance and sickness absence: a prospective study of employees working with clients. Int Arch Occup Environ Health, 2016; DOI 10.1007/s00420-016-1176-9.

Kahn RL, Wolfe DM, Quinn RP, Snoek JD, & Rosenthal R. Organizational stress: Studies in role conflict and ambiguity. 1964; New York: Wiley.

Karasek RA. Job demands, job decision latitude and mental health. Implications for job redesign, Administrative Science Quarterly, (1979;24:285–308.

Karasek RA, Brisson C, Kawakami N, Houtman I, Bongers P, Amick B. The Job Content

Questionnaire (JCQ): An instrument for Internationally Comparative Assessment of Psychosocial Job Characteristics. Journal of Occupational Health Psychology, 1998;3:322-355.

Kivimäki M., and co-workers (the IPD-Consortium). Job strain as a risk factor for coronary heart disease: a collaborative meta-analysis of individual participant data. Lancet, 2012;380:1491-1497.

Knardahl S. The health and well-being of healthcare workers. Concepts, theories and key work factors. In: L.T. Løvseth & A.H. De Lange (eds), Integrating the organization of health services, worker wellbeing and quality of care. Springer Nature Switzerland AG, 2020; doi.org/10.1007/978-3-030-59467-1_3.

Knardahl S, Johannessen HA, Sterud T, Härmä M, Rugulies R, Seitsamo J, Borg W. The contribution from psychological, social, and organizational work factors to risk of disability retirement: a systematic review with meta-analyses. BMC Public Health. 2017; Feb 8;17(1):176. doi: 10.1186/s12889-017-4059-4.

North FM, Syme LS, Feeney A, Shipley M, Marmot M. Psychosocial work environment and sickness absence among British civil servants: The Whitehall II study, American Journal of Public Health, 1996;86(3):332-340.

Rizzo JR, House RJ, & Lirtzman SI. Role Conflict and Ambiguity in Complex Organizations. Administrative Science Quarterly, 1970;15(2),150-163. doi:10.2307/2391486

Theorell T, Hammarström A, Aronson G, Träskman Bendz L, Grape T, Högstedt Ch. Marteinsdottir I, Skoog I, Hall Ch. A systematic review including meta-analysis of work environment and depressive symptoms, BMC Public Health*,* 2015;15:738.

Thoits PA. Mechanisms linking social ties and support to physical and mental health. Journal of Health and Social Behaviour, 2011;52:145-161.
